# Digital Fabrication and Theater: Developing Social Skills in Young Adults With Autism Spectrum Disorder

**DOI:** 10.3389/fpsyg.2021.615786

**Published:** 2021-03-09

**Authors:** Alicia Sandoval Poveda, Diana Hernández Montoya

**Affiliations:** ^1^Department of Special Education, School of Educational Sciences, Vice Presidency of Academic Affairs, Universidad Estatal a Distancia, San José, Costa Rica; ^2^Fabrication Laboratory Kä Träre, Vice Presidency of Research, Universidad Estatal a Distancia, San José, Costa Rica

**Keywords:** autism spectrum disorder, social skills, digital fabrication, scratch, theater

## Abstract

An action research project was carried out, using theater workshops and basic digital fabrication technology workshops to improve social skills—such as the expression of emotions, communication, self-control, and teamwork—in a group of 10 young individuals with autism spectrum disorder (ASD). This article focuses on the digital fabrication workshops, where participants worked on the fundamentals of electronics and programming, as well as 3D design and printing, to make props that were later used on stage in the theatrical performances in which they participated. These workshops were systematized through observation guides. According to the results, it was evident that the participants not only enjoyed and gained technological knowledge, but that their social skill development needs were addressed. Professionals from the Special Education Faculty and the Fab Lab Kä Träre, both from Universidad Estatal a Distancia in Costa Rica, were in charge of the study during its first phase, executed in 2016 and 2017.

## Introduction

Persons with autism spectrum disorder (ASD) have difficulty in developing social skills, an impairment that continues throughout their lives. According to the Organización Mundial de la Salud (2019), one out of every 160 children in the world has an ASD that persists into adulthood. This deficit affects them in different ways and, depending on each individual, impairs their functioning. Furthermore, due to their condition, individuals with ASD often experience stigmatization, discrimination, and inadequate access to services.

During their adolescence and young adulthood, individuals with ASD need support resources to help them develop new skills, a need that arises from the limited opportunities available to them to relate to peers outside the educational system and to access employment opportunities (Neary et al., 2015).

This paper describes the experience of a theater project for the development of social skills in people with ASD, that integrates elements of technology from a digital fabrication space. For this purpose, a review of previous works where art, technology and attention to disability are combined is made, to subsequently expose the work methodology used and the results achieved.

First of all, the use of theater to improve social skills is explored, as it has been documented that it is a practice that can enhance the behavioral and social environment of young adults with ASD (Corbett et al., 2016). Some of the best known theater projects used with this population are the Shakespeare Heartbeat project (Mehling et al., 2016; Loftis, 2019), the Miracle Project (Kim and Boyns, 2015; Feinstein, 2016; Ferguson, 2019) and the SENSE Theater approach (Corbett et al., 2014, 2019). In Costa Rica Universidad Estatal a Distancia (UNED) engaged in a similar project which resulted in the creation of the Rompecabezas Theater Group. This project was created based on the idea that theater, together with digital fabrication, was not only an option to work on social skill development, but could also be used as an educational and recreational space beyond the formal educational system, since most of the participants had already finished or were close to finishing their secondary education.

The project from which the Rompecabezas Theater Group emerged had the objective of helping participants develop some social skills by working with both theater and technology. The first of these skills, expressing emotions, was understood as the capacity of appropriately conveying to others what is being experienced by the self. The second skill, communication, was approached considering all components, from the explanations provided by the workshop facilitators to the questions asked by the participants. The third skill, self-control, especially self-regulation, was understood as the need to concentrate on the task at hand and to manage frustration throughout the different phases of the process and errors encountered. The fourth and last skill, teamwork, was identified as the capacity to work with other people in order to achieve a goal.

Rather than working from the perspective of what a person with a disability lacks, there are proponents who seek to work with these people’s talents and their development. This is Smagorinsky’s (2016) position, who states that young people with autism require the context to be adapted or constructed for them to make their greatest contributions. Among his proposals, this author emphasizes that theater and drama are an option for people with autism to relate to others with or without such condition and to themselves and their own needs. Interpreting other personalities allows for the construction and manifestation of one’s own personality which could otherwise remain unexpressed due to lack of context or the lack of possibility to do so.

According to Sherratt and Peter (2002), who were among the first to propose this type of intervention and on whom later interventions have been based, theater as art allows expressing meanings and feelings to an audience, as well as recognizing the fact that the world is ever changing, diverse and unpredictable. Furthermore, theater performance helps develop other skills like patience, confidence, control, self-exploration, language sharing, personal space sharing, respecting other’s time, and others.

Under this premise of using theater for youth with ASD to work on their social skills, several interventions have been implemented in educational centers (Kempe and Tissot, 2012; Neufeld, 2012; Calafat-Selma et al., 2016; Rivas, 2016) and groups created to work in theater (Casado, 2013; Guli et al., 2013; Kempe, 2014; Blanco et al., 2016). These experiences have been specific, have taken place in a reduced number of contexts, and have not been replicated.

Nonetheless, three large theater projects that have worked with individuals with ASD to develop their social skills served as the background to this project. These projects included Sherratt and Peter’s (2002) proposals in their references. The oldest is The Miracle Project, which uses a format of 22 sessions or one camping week, with the participation of people with ASD and the support of non-diagnosed young individuals; their families are involved in the logistics of presenting a musical show at the end of the process (Kim and Boyns, 2015; The Miracle Project, 2017).

The second, the Shakespeare Heartbeat approach, consists of 10 sessions and does not include a final presentation of a play, but uses acting methods, volunteer support and Shakespeare’s texts to play games (Mehling et al., 2016). Finally, the SENSE Theater utilizes several formats (3 months, 2 weeks camps, or 10 intensive sessions) ending with two public presentations of a theatrical play after engaging in theater games and acting exercises (Corbett et al., 2014).

All these projects are geared toward young individuals, do not have continuity after the program sessions conclude, and do not provide any instruction in or support with technology to the young participants to prepare for their performances. Overall, the use of technology in theater is infrequent, but it can be used to offer a modern and innovative perspective about theater (Sánchez et al., 2014).

In everyday practice, it is evident that people with disabilities often experience exclusion from adult educational opportunities and, especially, from the possibility of exploring artistic interests (Delgado and Humm-Delgado, 2017) even though Article 30 of the Convention on the Rights of Persons with Disabilities, ratified by Costa Rica by Law 8661 (Asamblea Legislativa de la República de Costa Rica, 2008), includes their right to develop and utilize their creative and artistic potential. This is addressed by the Rompecabezas Group’s proposal, which does not remain only in the artistic aspect, but also bets on the development of social skills through the overall experience in the theater and in the field of manufacturing.

Just like theater, technology is often used to help people with ASD in different environments (Cuesta and Abella, 2012); however, they are not usually provided with spaces to explore technologies like the ones used for digital fabrication on their own. In general, using this type of technologies enables the development of a sense of independence and creativity in the people that interact with them and motivates them to solve their problems and even tackle their own needs and the needs of other individuals.

On the other hand, García (2016) explains that fabrication spaces, like makerspaces, hackerspaces, fab labs, among others, have the common characteristic of acting as catalysts for encounters, production, and socialization; they all function as stimuli to their users, enabling them to establish relationships beyond the space itself, which was one of the objectives of this project.

The combination of art and technology is a trend that has been incorporated in different domains, including the STEAM (Science, Technology, Engineering, Arts, Mathematics) approach to learning. Within this new approach, the maker movement, from which fabrication labs derive, can play a fundamental role in the interaction among arts, science, and technology (Cilleruelo and Zubiaga, 2014).

UNED has a fabrication laboratory called Fab Lab Kä Träre, an open space that promotes empowerment by taking ownership of open technologies (Red de investigación, 2018) and other tools used in digital fabrication. Fabrication laboratories, like UNED’s, are part of the maker movement which promotes the creation of informal learning options that influence learning spaces by promoting their visitors’ active participation (Rosenfeld and Sheridan, 2014). The participation of the Fab Lab Kä Träre in the project “Developing Social Skills in People with ASD through an Artistic Experience: Theater” opens the possibility of integrating workshops on digital fabrication technologies into the initial project proposal.

Prior experiences have integrated technologies and programming in the work done with persons with disabilities. For instance, Scratch is a programming language that allows accessing programming from a basic level in a playful, significant, and social manner. According to different experiences (López-Escribano and Sánchez-Montoya, 2012; Munoz et al., 2016; Gribble et al., 2017), this program has been used to work with persons with disabilities, including people with ASD. Learning through a program like Scratch is accomplished via problem-solving by discovering solutions at each person’s own pace (Sáez-López et al., 2016). In fact, the program is designed in such a way that all people, regardless of age or condition, can use it (Marcelino et al., 2018).

Although there could be real difficulties to teach these new technologies to these populations, persons with disabilities have a right to learn about them. According to the United Nations Convention on the Rights of Persons with Disabilities (Organización de Naciones Unidas, 2007), they have the same right to have access to education and information technologies as people without disabilities. Similarly, the Convention upholds their right to access all recreational opportunities and to develop their creative and intellectual potential. However, according to the Organización Mundial de la Salud (2011) most recent report on disabilities, persons with disabilities are less likely to access technology and information than those who do not have any disability.

According to Quirós (2017), progress has been made in Costa Rica as projects, both public and private, strive to improve the access of persons with disabilities to information and communication technologies, but, in general, limitations to access cell phones and computers still exist. According to this author, inclusion regarding the use of technology required not only access, but also empowerment for the people with disabilities to be able to use different types of technologies. More openness is needed to bring such knowledge to that particular population.

Graphic programming environments have become an affordable option to teach programming in a straightforward manner. They are popular because they make programming languages available in an easy-to-understand way, especially block-based languages which omit programming syntax and focus on creation (Hill, 2015).

Graphic- and block-based programs in environments like Scratch have proven accessible to working with persons with special educational needs, such as intellectual disabilities, autism, motor disabilities, and visual disabilities (López-Escribano and Sánchez-Montoya, 2012).

At the beginning, Scratch’s goal was to bring programming to people who have never viewed themselves as programmers, so that they could promote their creativity and expression through this activity. To accomplish this, the approach focused on overcoming the difficulties of previous programming teaching methods and became more customizable, meaningful, and social than other programming environments. That is why it is characterized by its ease of use (which allows individuals to develop their own projects) and by making a webpage available to be used as a platform to share projects with others and discuss their development (Resnick et al., 2009).

López-Escribano and Sánchez-Montoya (2012) cite two experiences of people diagnosed with autism using this type of tools. The first is Adams (2010) who compiled his Scratch programming camp experiences for young persons with ASD. He reports on a 14 years old boy with high functioning autism who had verbal outbursts when he got excited or frustrated yet was able to create his own video game during the camp.

The second experience is from Vicki Gold at a high school where she has been using Scratch to work with young students with Asperger since 2006. Her compilation of experiences states the importance of providing individualized supervision to each student instead of just guiding them with highly structured classes that do not attract their attention (Scratched Team, 2011).

Vallejo (2018) adds that the possibility of creating through programming can also promote innovation. Providing opportunities for creation, collaboration, and innovation is also part of what is promoted by the maker movement, where “doing” is a way of learning (Rosenfeld and Sheridan, 2014). This is how co-creation spaces, like the fabrication laboratories located in formal or informal educational environments, seek to empower individuals not only to use technology, but also to build it (Blikstein and and Krannich, 2013). It is important to emphasize one of the primary characteristics of the fabrication laboratories: that of being open to any person. García (2016) explains that they are available as community resources that provide open access to individuals to develop programs and projects.

Objectives like “co-creation,” “empowerment,” and “knowledge sharing” could create reluctance toward using these kinds of approaches with persons with ASD because of the basic criteria to diagnose this condition: persistent deficits in social communication and interaction across multiple contexts; restrictive patterns of behavior, interests, or activities (American Psychiatric Association, 2014).

Additionally, the interventions for the ASD population usually prioritize objectives related to improving communication skills and making their behavior more flexible, as well as regulation of emotions and self-control (Martos-Pérez and Llorente-Comí, 2013). These social skills were contemplated in the Rompecabezas Theater Group project and were tackled not only from the theater stages, but also from the use of technologies in the Fab Lab Kä Träre. The activities included in both spaces allow them to learn and perform in environments different from their daily spaces, work in teams to set up a play, solve exercises or build materials for the play, face frustration when a scene or a program did not work as expected, experience many other situations that came along and became opportunities to improve their social skills.

It was precisely the combination of all the opportunities and experiences provided by their participation in the theater and the Fab Lab Kä Träre which allows the members of the Rompecabezas Theater Group to be the main characters in a play and to create and share their knowledge about new technologies, but, above all, allows them to develop new social skills to perform better in all areas of their lives.

## Materials and Methods

### Approach

The project started in March 2016 and used a qualitative approach with an action research methodology (Hernández et al., 2010) and with the involvement of UNED (through its Department of Special Education and the Fabrication Laboratory Fab Lab Kä Träre), La Máscara Theater, and the persons with ASD and their families.

The first phase of the project lasted 2 years, 2016–2017, during which the same group of young individuals with ASD participated in theater workshops and three technology workshops. The theater workshops continued for an additional year with the performance of a new play by the original group. A new group received acting lessons as well.

A group made up by three professionals in special education, a language therapist, an educator and actress, and a psychologist were in charge of the research in the theater. In turn, the Fab Lab Kä Träre staff was in charge of developing the first technology workshop and providing assistance and support for the second and the third workshops. To do this, the research group trained the laboratory staff in ASD, who, in turn, participated in several training sessions in electronics, 3D modeling and programming.

### Analysis Units

The group started with 10 young participants with ASD—3 women and 7 men—with an average age of 17.01 (*SD* = 4.01), who lived in the Great Metropolitan Area. One of them was attending elementary school (12 years old), 8 were in high school, and one had completed secondary school. The inclusion criteria required all of them to have been diagnosed with ASD by a specialist who had worked with them in the past; they had to be between 12 and 20 years old; and all needed to work on improving their social skills. The exclusion criterion was not being able to work independently from a caretaker or support teacher. The invitation to participate in the project was made first through professionals working with persons with ASD, and then by disseminating information about the project in the mass media.

All participants or their legal guardians signed the research informed consent approved by UNED’s Vice Presidency of Research, under registration code PROY0048-2016, which included the theater workshops and the technology sessions.

**FIGURE 1:**
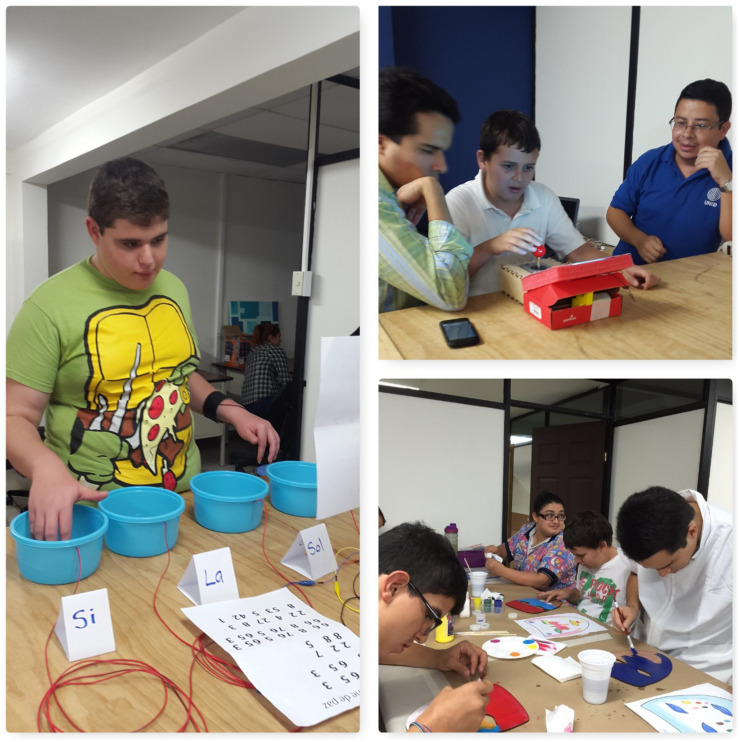
Workshop participants experiment with Makey-Makey and make their own masks with lights. This image is for the exclusive use for the purposes of this research.

**FIGURE 2:**
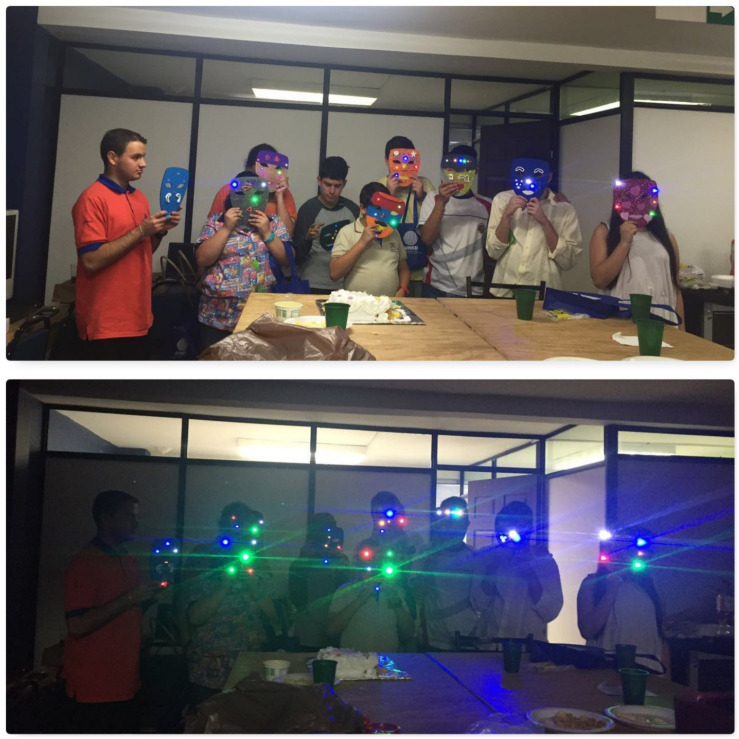
Workshop participants show off their finished masks with lights. This image is for the exclusive use for the purposes of this research.

**FIGURE 3:**
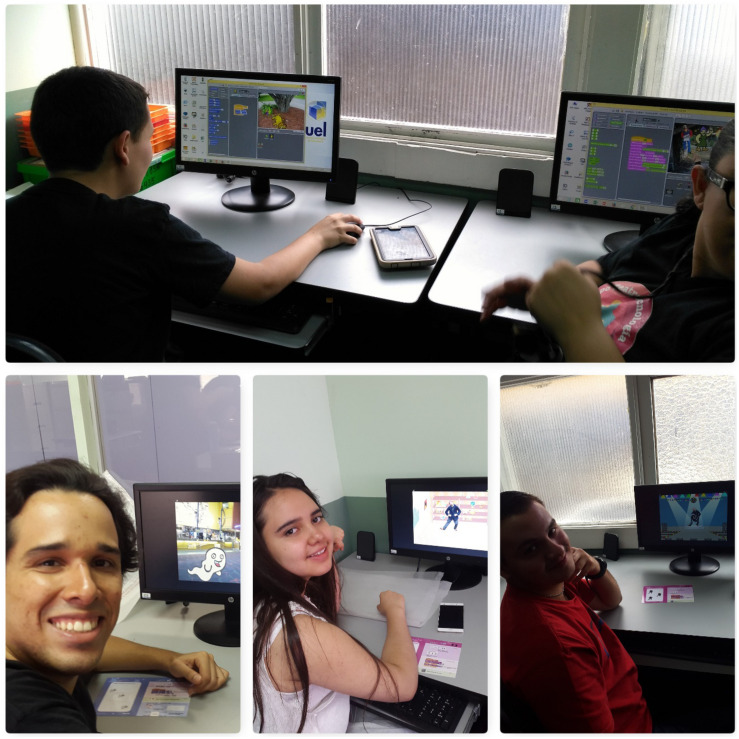
Participants in the Scratch programming workshop. This image is for the exclusive use for the purposes of this research.

Once the group was complete, the theater workshops started with weekly one-and-a-half-hour sessions at La Máscara Theater. During 2016, they took acting classes and performed in a play as actors and actresses at the end of the year. In 2017, two productions were presented, which increased the complexity for the youth with ASD. In other words, one theatrical play was produced in 2016 and two in 2017. Before each of the performances, a technology workshop—for a total of three—was organized:

• Workshop on basic digital fabrication: it took place at the Fab Lab Kä Träre between May and June 2016. Prior to this workshop, the families participating in the project had the opportunity to visit the place and see the technologies the young individuals would be utilizing. The workshop consisted of eight 1 h work sessions filled with playful activities to approach concepts like circuits, resistors, 3D printing, 3D modeling, as well as creation activities, among others. Other technologies included Makey-Makey, led lights, lithium batteries, jumper cables, copper tape, and other electronic materials (Figure 1). Each hour-long session was dedicated to a specific activity and guide. Four facilitators from the laboratory planned and directed the experience. The workshop’s objective was to create laser cut masks, personalized with lights and 3D printed elements to use them in their first theatrical performance (Figure 2).

• Workshop on Scratch programming: This workshop was taught at the computer lab of UNED’s University Center in San Jose between February and March 2017. It consisted of seven 1 h work sessions where the participants learned the core principles of Scratch programming. Each participant worked individually in a computer to generate his or her own animations following working guides to learn how to make animations provided by the Scratch platform itself (Figure 3). Each one developed the guides at their own pace throughout the hour-long session, until, for those who were able, they generated their own animation. The sessions were taught by three facilitators: a special education teacher, a language therapist, and a psychologist that had been trained by the Fab Lab Kä Träre staff. This workshop’s objective was for each student to make an animation of animals interacting with each other inspired in the production they would be putting on stage in June 2017, where all characters were animals in a planet threatened by pollution.

• Workshop on mBlock programming: it was held in November 2017 and took a 3 h session where participants used the knowledge acquired in the workshop on Scratch to program and personalize the illumination of a Christmas lamp that they had to use in the third play where they portrayed the inhabitants of a town going out at night looking for invaders that came to disturb their Christmas. A Digispark ATTINY85 board for Arduino IDE was also used. The materials and the programming were planned in advance in the Fab Lab Kä Träre, where the lamps were designed and the type of circuits and programming to be used was selected (Figure 5). The workshop was taught by two of the project researchers, a special education teacher and a psychologist, with the support of two volunteers, professionals in education, in a classroom of the University Center in San José.

In order to carry out the workshops with this specific group, it was necessary to make some modifications with respect to how these types of spaces are usually taught. First, the Fab Lab’s staff conducted a training and awareness session on ASD, to learn about the characteristics of the population and unlearn myths about what to expect from the group. Secondly, a previous session was held with the mothers of the participants so that they could get to know the fabrication lab space and the facilitators.

For the execution of all the workshops, detailed work guides were developed, which were presented in three different formats, appealing to the different ways of communication so that each participant could take advantage of the one they understood best: verbal instructions, a printed sheet with written instructions and photographs, and the projection of the instructions on the blackboard. The Scratch guides that were specific to the program were not modified but were delivered to them in the same way by different means. In addition, there were four facilitators per session, a larger number than usual, in order to provide more personalized attention and reduce waiting times. The profile of the facilitators was also varied, given that a professional in psychology or education was always present along with the laboratory staff, in addition to the support of other special education teachers.

Efforts were also made to reduce distractions in the space to avoid visual and auditory overstimulation. For example, no other computers were on and no 3D printing unrelated to the workshop was performed while the workshop was in progress. Finally, it is important to consider that entire sessions were devoted to tasks that in other groups would have been only part of the session, for example, learning how to build a circuit or following an animation guide in Scratch.

### Data Gathering Techniques

The youngsters’ progress during the project was recorded through observations and field logs. In the specific case of the activities that were held using technological resources, three instruments were used:

• A work log for the Fab Lab Kä Träre sessions: a narrative was recorded at the end of each session on the log, which detailed each participant’s performance, behavior, and comments during the session.
• A compliance log for the Scratch activities and skills: the facilitators used the log to record the tasks completed by the participants and their behavior during the sessions using a checklist to show whether or not they completed the activities.
• Photo journal: it consisted of a set of photos and videos of the projects that integrated different technologies created in the Fab Lab Kä Träre workshops by the participants.

The three instruments included categories that were operationalized to have a clear understanding of what behaviors were indicators for each of the aspects under study: self-control, expression of emotions, communication, and teamwork.

### Analysis

A content analysis of the photos and logs was performed to classify them according to the previously defined categories in order to assess how the technology work sessions had contributed to the development of the participants’ social skills. These categories are the following:

• Expression of emotions: verbal and non-verbal manifestations of how the participants felt toward the exercises, whether these expressions targeted their peers or the facilitators.
• Communication: verbal expression of ideas, opinions, or questions addressed to their peers or the facilitators, either about the technology they were working with or about personal matters.
• Self-control: frustration management to deal with errors, on-task behavior, and control of anxiety when facing new activities.
• Teamwork: providing or requesting assistance from peers to conclude the tasks assigned.

The work log was read to separate each behavior and comment recorded according to the four work categories. Then, the content units for each category were grouped to discuss, based on the theory, the progress observed in each area. The photos and compliance logs served as supplement to triangulate the young individuals’ participation during the workshops.

## Results

After the technology workshops organized in the first 2 years of the project, it was possible to identify a series of behaviors related to each of the social skill categories defined in the project objectives. Progress identified through content analysis of photographs and logs is presented below. First, the results in the fabrication workshops, classified according to categories, and second, other progress identified in the subsequent programming workshops.

**FIGURE 4:**
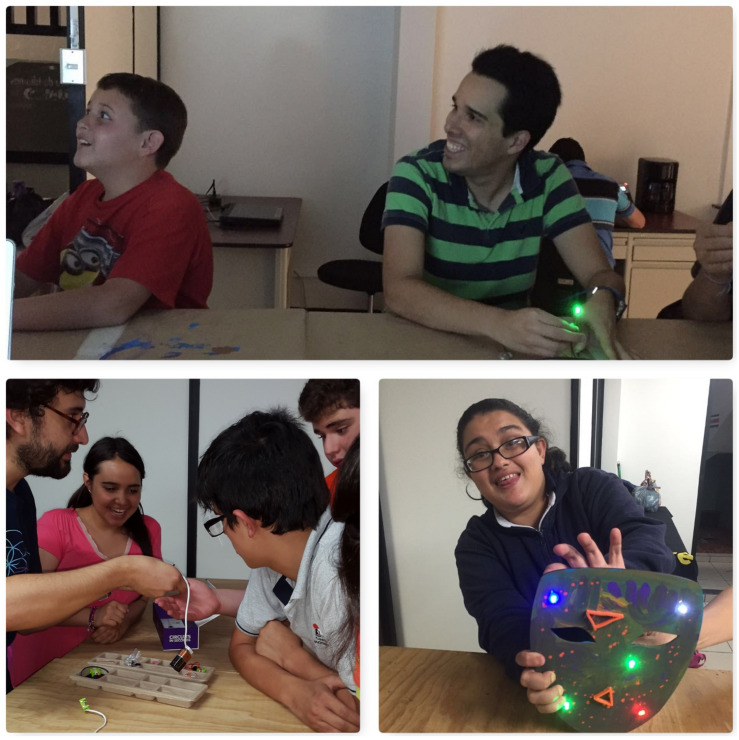
Expression of emotions of the participants during the basic digital fabrication workshop. This image is for the exclusive use for the purposes of this research.

Regarding the expression of emotions, all the participants expressed at some point after the workshops had started, either verbally or non-verbally, how they felt with the activities at hand: happy for the results accomplished or sad and upset about the difficulties encountered. As the sessions went on, the expressions of emotions related to the fabrication work performed increased. Below are some examples of behaviors for this category (Figure 4).

**FIGURE 5:**
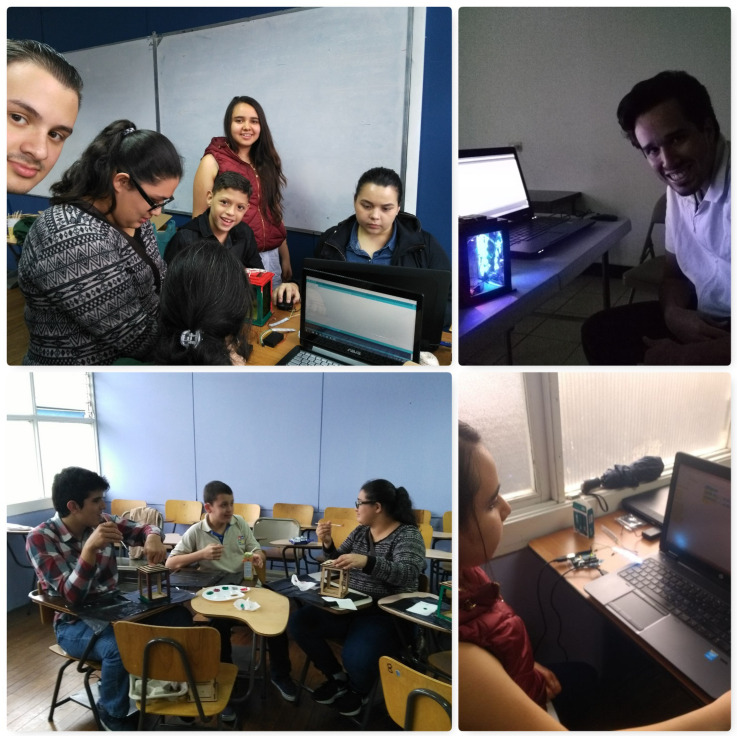
Participants in the mBlock programming workshop. This image is for the exclusive use for the purposes of this research.

When working with LittleBits: one of the participants became excited with the blocks. He really liked comparing them with Legos because he said he had 35 boxes of Legos at home. He constructed a whole circuit with them (Laboratorio de fabricación Kä Träre, 2016).

On the last day of the lab activities, one of the participants painted a butterfly like the one she had made for the mask and said that what she had liked the most was 3D printing. Then, she hugged everyone good-bye (Fab Lab Kä Träre, 2016).

In turn, communication skills were assessed in two areas: interaction among the participants and facilitators and interaction among the participants themselves. At the beginning of the process, the relationship with the facilitators solely involved asking questions about how to complete a task, but as the sessions progressed, they queried about more personal topics. Although at the beginning they were silent, as the sessions progressed, the frequency with which they talked to each other about what they were doing increased.

In summary, there were improvements in how to ask questions to the facilitators, how to talk and comment on personal issues, and how to communicate with other people who came to the lab even if they were not part of the workshop. Some examples of behaviors in this category are the following.

When the Makey-Makey board was shown to them, several participants asked how it worked (Fab Lab Kä Träre, 2016).

While creating a basic electrical circuit, one of the participants frequently interacted with one of the lab facilitators by asking questions and calling for help. This participant talked a lot while developing the circuit (Fab Lab Kä Träre, 2016).

The behaviors observed related to self-control were diverse. Some were necessary to perform the activities, like waiting for one’s turn, following directions, and persevering in their attempts to complete the tasks assigned, without presenting behavioral outbursts like crying, complaining, or yelling. At the beginning it was more frequent to observe frustration behaviors (giving up on the work, demanding attention, moving forward without waiting for instructions) and as the sessions went on it was more frequent for them to wait their turn, follow verbal instructions to carry out the activities and ask for help to carry out the work.

Other self-control behaviors were related to the participants’ behavior during the sessions. There were participants who at the beginning interrupted with topics of their interest outside the workshop, but as the sessions went on, they interrupted less with other topics during the work sessions. Also, some learned to like not talking about unrelated topics, self-directing, and tolerating annoying environmental distractions, like the noise in the lab (3D printers and computers) that initially caused the most discomfort. In specific cases, improvements were made in regulating what topics they could talk about (such as not asking invasive personal questions) or removing personal items that made them feel safe to be in the space (such as one young woman who by the fourth session removed a purse that she never left).

It was also possible to see that each participant’s concentration span on the tasks at hand increased session after session. In the first sessions, activities took 10 min, while by the end, they were able to focus on a single 40 min activity. Teamwork was encouraged; this made it possible for them to share, in several sessions, materials to complete their tasks and work in groups of up to four people per table. In the last session, they did not have an activity structured for them in advance; instead, they were asked to choose what to do, regulating their own work process for about half-an-hour which would not have been possible at the beginning of the process.

Some examples of behaviors for this category are the following.

During one of the technology exercises circuits, one of the participants wanted to do a different activity before working on the mask. So, he was told that this could only be possible after the mask was completed which caused him some anxiety (Fab Lab Kä Träre, 2016).

One of the highlights of the process was the free activity where they were asked to create a collage with different materials; the fact that they did not destabilize when asked to do something on their own was positive. At the beginning of the process (the workshops) they had to be told what to do step by step; however, at this time the group evidenced their empowerment regarding the space and the activity. The teamwork category was the most complicated to observe due to the fact that tasks were to be performed individually for the most part or at least under individualized attention. However, they also worked in pairs in a more or less successful way; they learned to correct each other when they were doing something wrong, to explain to each other how to do something, and to share materials to carry out a task.

**FIGURE 6:**
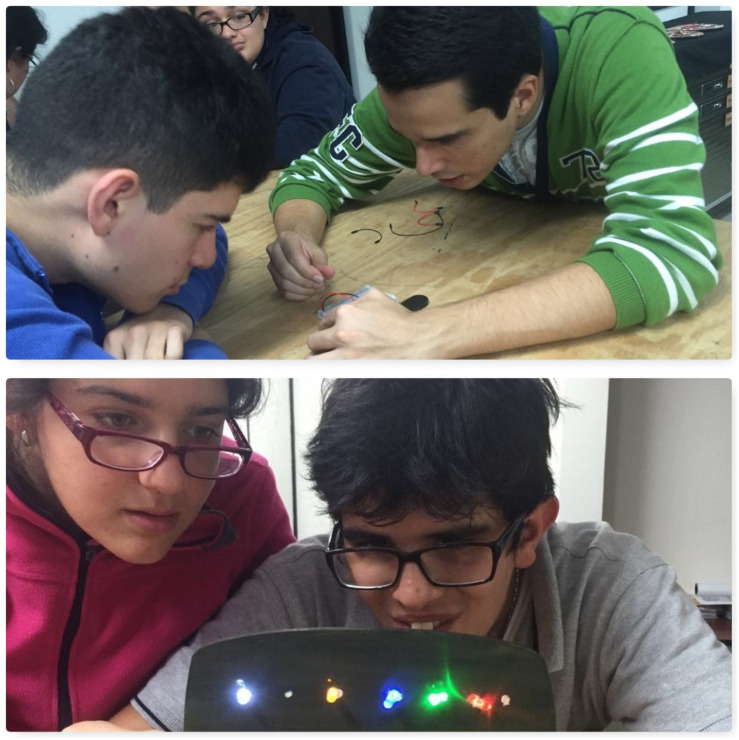
Examples of teamwork during the basic digital fabrication workshop. This image is for the exclusive use for the purposes of this research.

As for teamwork, it was enhanced by making it possible, in several of the sessions, to share materials to complete the tasks, as well as sharing work tables among up to four people. This helped them learn to share materials even though at the beginning they asked to have their own. They also developed exercises together in pairs, something they had not done before, and developed skills to correct or explain to the person next to them what they were doing (Figure 6).

All behaviors observed in the three technology workshops are summarized and classified by category in Table 1.

**TABLE 1:** Behaviors recorded in the technology work sessions by category.

**Category**	**Behaviors observed that appeared during the course of the technology workshops**
Emotions	Smiling and making comments of joy when completing exercises
	Asking for forgiveness when they were wrong
	Manifest that they were sad because the workshops were over
	Laughing and clapping during a social sharing activity held at the end of the process
	Formulating complaints about the difficulties they encountered in any exercise
Communication	Asking the facilitators for help
	Inviting others to see the result of their work
	Asking the facilitators questions about the exercises
	Asking peers how they did something
	Asking for more information about the technology they were using
	Asking the facilitators personal questions
	Commenting about their daily activities
	Taking the initiative to speak with visitors (outsiders) who came to the workshops
Self-control	Reducing tantrums related to the exercises or activities
	Increasing the on-task concentration spam
	Repeating tasks until accomplishing the expected result
	Waiting for their turn when working in groups or asking questions
	Following oral instructions about their work
	Following a work guide
	Continuing with the work in spite of intense noises
	Leaving personal questions or games to the end in order to focus on the task at hand
	Getting away from objects that gave them confidence (e.g., a participant was able to let go of her purse in the last sessions to do her work)
	Giving directions to themselves aloud (e.g., “I will not go on with this topic.”)
	Choosing a free activity to do without guidance
Teamwork	Completing exercises in pairs
	Correcting peers when they made mistakes in the exercises
	Sharing materials with peers to complete the tasks
	Explaining to their peers how a task had to be done

*Proprietary elaboration.*

During the sessions in electronics and 3D printing, the participants’ progress was observed with respect to how they asked the facilitators questions, how they spoke about and commented on personal topics, how they communicated with lab visitors, how they expressed joy when a task was completed, how they followed oral directions to do what was required and, in specific cases, how they controlled what topics to talk about and let go of personal objects which had usually given them confidence (e.g., the young girl that by the fourth session let go of her purse, something she had not done before).

New progress was evidenced in the Scratch work sessions and the generalization of behaviors learned in the previous workshop was observed. Some of the participants had to learn to express their discomfort with the exercises that were difficult for them, without an emotional outburst; while others expressed their joy through laughter and comments whenever they completed a programming task. Regarding communication, they replicated appropriate forms of attracting others’ attention and asking for help that they had started to use in the previous workshop. Additionally, they worked on self-control by taking turns to receive personalized attention. Regarding this category, it should be pointed out that working with Scratch allowed each of the participants to follow a programming work guide and advance at their own pace promoting self-regulation.

Since each participant was working with one computer and his or her own specific programming guide, there were fewer possibilities to work in teams. Sometimes, however, when directed by the facilitators or upon the initiative of a classmate, some of those who were more advanced approached others to explain how they had done the task.

Finally, in the session to make and program the lamp, priority was given to working on self-control and in teams. Each participant was assigned a specific time to program and practice the skills of following directions and taking turns to work on their program. While they were waiting, they had to work in sub-groups sharing the materials to paint and make their lamps. Thus, it was possible to observe, throughout the 16 sessions, the participants’ progress in the four categories analyzed.

The outputs from each of the technology workshops are presented in Table 2.

**TABLE 2:** Outputs of the technology workshops.

**Workshop**	**Number of participants**	**Outputs**
Basic electronics	10	Nine masks with five led lights in a circuit and two 3D-printed accessories as ornaments
Scratch	8	Three complete, animated scenarios
		Three unfinished scenarios
Lamps	6	Six customized painted lamps with programmed lighting effects

*Proprietary elaboration.*

One of the participants left the project before finishing the basic electronics workshop; therefore, only nine participants finished their mask. In 2017, eight individuals participated in the Scratch workshop, but only six were able to make the animation, three of them actually completed it. The lamp workshop was voluntary, and only six of the group members participated; they all finished the lamps with all the details: customized painting and lighting effects.

## Discussion

Developing social skills is one of the main objectives when seeking to improve the quality of life of persons with ASD. Theater has become an option to work on these skills. Therefore, based on this premise, a project was developed at UNED which led to the creation of the Rompecabezas Theater Group.

As has already been mentioned, this project’s objective was to improve the social skills of youth with ASD, using not only theatrical plays, but also other novel options, like the Fab Lab Kä Träre. Then came the challenge of working on the same skills that were to be improved using theater (expression of emotions, communication, self-control, and teamwork) through experiences in other areas that are not easily accessed by this population, such as those that offer the possibility of interacting with digital fabrication technologies.

This 16-session experience brought about results that make us think that working with young persons with ASD using these technologies is also a way of promoting social skill development and that they have great potential in areas that this population had not accessed before: programming, 3D printing, basic electronics.

This research is considered to have provided a valuable contribution. Most of the other interventions are concrete programs for populations with ASD developed in clinical or academic contexts that usually use classical behavioral and cognitive techniques (Martos-Pérez and Llorente-Comí, 2013) or focus on different fields or techniques, like soft skills for daily life, referential communication, agendas, social stories, and others (Olivar and de la Iglesia, 2009).

People with ASD vary in terms of the difficulties they face; therefore, it is not easy to generalize what type of interventions are more suitable for them. Although there are clinical interventions that have proven efficient (often considered to be evidence-based), it is methodologically difficult to verify the efficacy of all interventions. Many of them are designed to be used at a clinical or academic level, but not at a community or everyday level (McLeod et al., 2015).

This research proposes combining theater (Sherratt and Peter, 2002; Corbett et al., 2014; Kim and Boyns, 2015; Mehling et al., 2016) with the digital fabrication as an intervention for individuals with ASD, a novel approach not used in other theater-based intervention studies. There is no background work in basic electronics, 3D printing, and others. Regarding fabrication labs and block programming environments, there have only been two specific experiences using Scratch with individuals with autism (López-Escribano and Sánchez-Montoya, 2012).

This situation evidences the novelty of the proposal that was successfully put into practice, which, through observation and logs, allowed identifying behaviors that evidenced the participants’ progress in the skills under study. What this project proposes, then, is that it is indeed possible to open varied and challenging spaces for the youth with ASD to learn and interact with new technologies and, in turn, that the interaction with other people in a significant learning context like this one, favors the actual use of the social skills they are developing.

It has been evident that the specific features of a program like Scratch make it accessible to all people, beyond their skills to understand a programming language (Resnick et al., 2009) and that block programming environments increase the possibilities for people to access the benefits that studying programming create (Hill, 2015).

Technology is not foreign for people with ASD or other disability conditions. It is many times used as a facilitator to provide support tools for the person to face his or her difficulties (Cuesta and Abella, 2012). However, this proposal aims at giving them access to technology for their education and entertainment and, as evidenced, to improve their social skills at the same time. These opportunities are necessary because, as they move away from the formal educational system of their childhood and adolescence, people with ASD face difficulties to find this type of learning and workspaces (Neary et al., 2015); therefore, this could create a wealth of new possibilities.

To continue in this line of work, it is necessary to find new, more accurate approaches to assess the participants’ progress in the workshops, both in terms of their social skill development and their use of technology. It is necessary to replicate the experience of working with electronics and programming with more groups, more young individuals, and for a longer period of time in order to gather specific information that can guide the assessment of the intervention to determine its efficacy. The results of this study show that there is a possibility of working on these skills in this type of learning space, but further research is needed to pinpoint the relationship between the methods used and the progress observed.

Working with theater and technology allowed bringing art and technology together to provide opportunities for young people with ASD who found in this combination a space of interest for recreation and learning. Demonstrating the possibility of implementing this proposal and observing progress in the participants is a first step already taken by this study.

Learning about digital fabrication technologies – electronics, programming, and 3D printing – opened a space where the participants were able to express their joy for the good results and their sadness and concerns for the difficulties faced. They were able to communicate with the facilitators and the other group members. The program also facilitated the development of self-control strategies like following directions, taking turns, managing frustration, and persevering until the task was completed. It opened up teamwork spaces where they were able to share and help each other. All this was recorded through observations and photo journals throughout the 16 working sessions.

Although this is not the first research using theater for social skill development, it is novel in its use of technology as part of the process. Based on the results of this experience, it is convenient to develop more research proposals along this line, either theater integrating technology or technology workshops designed for persons with ASD. The approach could even be expanded to include people with other disability conditions that could present similar needs.

## Data Availability Statement

The raw data supporting the conclusions of this article will be made available by the authors, without undue reservation.

## Ethics Statement

The studies involving human participants were reviewed and approved by the UNED’s Vice Presidency of Research, under registration code PROY0048-2016. Written informed consent to participate in this study was provided by the participants, and/or where necessary, the participants’ legal guardian/next of kin. Written informed consent was obtained from the individual(s), and minor(s)’ legal guardian/next of kin, for the publication of any potentially identifiable images or data included in this article.

## Author Contributions

Both authors, from their areas of specialization and work at the University, participated in both the research and the elaboration of this article.

## Conflict of Interest

The authors declare that the research was conducted in the absence of any commercial or financial relationships that could be construed as a potential conflict of interest.
